# Ethnic heterogeneity and prostate cancer mortality in Hispanic/Latino men: a population-based study

**DOI:** 10.18632/oncotarget.19068

**Published:** 2017-07-06

**Authors:** Felix M. Chinea, Vivek N. Patel, Deukwoo Kwon, Narottam Lamichhane, Chris Lopez, Sanoj Punnen, Erin N. Kobetz, Matthew C. Abramowitz, Alan Pollack

**Affiliations:** ^1^ Department of Radiation Oncology, University of Miami, Miami, FL, USA; ^2^ Sylvester Comprehensive Cancer Center, University of Miami, Miami, FL, USA; ^3^ Department of Urology, University of Miami, Miami, FL, USA; ^4^ Division of Population Health and Computational Medicine, Department of Medicine, University of Miami, Miami, FL, USA

**Keywords:** prostate cancer, cancer specific mortality, Hispanic/Latino, health disparities, minority health

## Abstract

**Background:**

Few studies focus on prostate cancer (PCa) outcomes in Hispanic/Latino men. Our study explores whether Hispanic/Latino subgroups demonstrate significantly different prostate cancer-specific mortality (PCSM) relative to Non-Hispanic White (NHW) and Non-Hispanic Black (NHB) men.

**Methods:**

We extracted a population-based cohort of men diagnosed with local-regional PCa from 2000-2013 (n= 486,865). PCSM was measured in racial/ethnic groups: NHW (n=352,886), NHB (n= 70,983), Hispanic/Latino (n= 40,462), and Asian American/Pacific Islander (n= 22,534). PCSM was also measured in Hispanic/Latino subgroups: Mexican (n= 8,077), Puerto Rican (n= 1,284), South or Central American (n= 3,021), Cuban (n= 788), and Dominican (n= 300). We conducted univariable and multivariable analyses (MVA) to compare risk for PCSM.

**Results:**

Compared to NHW men, results showed worse outcomes for NHB men with similar outcomes for Hispanic/Latino men. In MVA with NHW men as a reference, NHB (HR= 1.15, *p* <0.001) men had significantly worse PCSM and Hispanic/Latino (HR= 1.02, *p*= 0.534) men did not show a significant difference. In a second MVA, Puerto Rican (HR= 1.71, *p* <0.001) and Mexican (HR= 1.21, *p*= 0.008) men had significantly higher PCSM. With NHB men as a reference, the MVA showed Puerto Rican (HR= 1.50, *p*= 0.006) men with higher PCSM and Mexican (HR= 1.08, *p*= 0.307) men with no significant difference.

**Conclusions:**

Our findings indicate previously unknown disparities in PCSM for Puerto Rican and Mexican American men.

## INTRODUCTION

Disparities in prostate cancer (PCa) treatment and outcome among Non-Hispanic Black (NHB) versus Non-Hispanic White (NHW) men have consistently been reported in the literature [1, 2]. Socioeconomic and cultural factors, such as insurance coverage [2] or modifiable patient-provider decisions [3], contribute to such disparities. Unique biological differences have been reported as well [4, 5]. Through careful consideration of potential influences of these disparities on disease incidence and outcomes, more exact conclusions can be made regarding the etiology. Currently, most inquiries focused on disparities fail to acknowledge important variability that occurs within racial and/or ethnic groups. Identifying such variability, should it exist, is essential for informing appropriate clinical screening and management of disease.

Individuals are typically assigned to a racial and/or ethnic group based on skin color [6], country of origin or ancestry, language or dialect spoken [7], and self-identification. According to the U.S. Census Bureau, Hispanic or Latino individuals in 2014 reached about 55.4 million or 17.4% of the national population, making them the largest ethnic minority group in the United States of America [8]. Recent reports show that incidence and death rates among Hispanic/Latino men diagnosed with PCa are slightly lower than that of NHW men with NHB men having the highest rates [9]. As described in prior publications, there is the potential to over-generalize cancer outcome data by aggregating all Hispanic/Latino subgroups into one broad category for research purposes [10, 11].

Attempts to codify racial and/or ethnic categories will inevitably group heterogeneous populations. Within the Hispanic/Latino community, 64.9% are Mexican, 9.2% are Puerto Rican, 3.7% are Cuban, 3.0% are Dominican, and 19.2% are from other Hispanic/Latino groups [12]. With racial and/or ethnic categories being broad and overlapping, there is potential to obscure significant within-group differences [7, 10]. Some studies have attempted to disaggregate ethnic subgroups by ancestry and/or geographic region of origin to highlight differences in health characteristics due to heterogeneity. A stroke study by Arauz et al [13] explored the difference in lipid profiles between a predominantly Caribbean-Hispanic/Latino population in Miami, FL, U.S.A. and a predominantly Mestizo-Hispanic/Latino population in Mexico City, Mexico, ultimately finding significant differences in lipid profiles due to Hispanic/Latino racial or ethnic heterogeneity. Similarly, a nationwide population-based study by Chao et al [14] features higher PCa-specific mortality (PCSM) rates in Hawaiian and Pacific Islander men compared to NHW and other Asian American subgroups. They describe the heterogeneity found within the Asian American/Pacific Islander (AAPI) group and that the results suggest the need for disaggregation of AAPI data. Potential underlying differences in PCSM amongst the genetically and culturally diverse Hispanic/Latino subgroups have yet to be adequately characterized and then investigated for influential factors.

Despite those reporting survival differences based on cost of care [15], immigration status, and living in ethnic enclaves [16], much more is needed to further understand the potential role of ancestry and/or geographic region of origin in contribution to PCa incidence and outcome within the Hispanic/Latino male population. Recent studies examining racial and/or ethnic influences on PCa, have focused on NHW and NHB men [17–19]. Among the few cancer-related articles discussing Hispanic/Latino subgroups, the use of state-specific cohorts may reduce their generalizability to the national population [20, 21]. To our knowledge, no other reports have addressed the heterogeneity within Hispanic/Latino men diagnosed with PCa from a national population-based cohort. Our study highlights the heterogeneity within Hispanic/Latino men and identifies populations that appear to be at greatest risk for mortality from PCa.

Our findings suggest that heterogeneity in PCSM exists within Hispanic/Latino men diagnosed with PCa in the United States. We identified two Hispanic/Latino subgroup populations with higher rates of PCSM after diagnosis with local-regional disease when compared to NHW men.

## RESULTS

In Table 1, the baseline characteristics of the patient cohort divided into racial and/or ethnic groups (NHW, NHB, Hispanic/Latino, and AAPI) are displayed. Significant differences were found across these groups in marital status, insurance status, TNM stage, socioeconomic status (SES) composite score, disease stage summary, grade, residence type, treatment type, and age at diagnosis. In Table 2 we show the baseline characteristics for NHW men and all Hispanic/Latino ethnic subgroups. Although significant differences were found across these groups in TNM stage, SES composite score, grade, residence type, and treatment type (*p* <0.001); the magnitude of such differences were relatively small. Moreover, Mexican Americans were more likely than all other subgroups to have regional disease (17.2% vs. 9.7-14.8%) and use any Medicaid insurance (16.2% vs. 8.2-14.3%), while Puerto Ricans were less likely to be married (59.3% vs. 65.3-73.9%), and Cuban Americans were more likely to have a higher age at diagnosis (69.8 years vs. 64.6-65.9 years).

**Table 1:** Patient, tumor, and treatment characteristics divided by racial and/or ethnic group

*Variable*	*All*	*Non-Hispanic White*	*Non-Hispanic Black*	*AAPI*	*Hispanic/Latino*^*a*^	*p-value*
	*N (%)*	*N (%)*	*N (%)*	*N (%)*	*N (%)*	
**Total patients**	486,865	352,886	70,983	22,534	40,462	
**Age at diagnosis**	66.0 ± 9.2	66.5 ± 9.2	63.5 ± 9.1	67.9 ± 9.0	65.7 ± 9.2	<0.001
**Marital status**						<0.001
*Married*	330,254 (67.8)	247,543 (70.1)	38,444 (54.2)	17,049 (75.7)	27,218 (67.3)	
*Others*	102,160 (21.0)	66,177 (18.8)	24,292 (34.2)	3,141 (13.9)	8,550 (21.1)	
*Unknown*	54,451 (11.2)	39,166 (11.1)	8,247 (11.6)	2,344 (10.4)	4,694 (11.6)	
**Insurance status**						<0.001
*Uninsured*	4,745 (1.0)	2,224 (0.6)	1,463 (2.1)	207 (0.9)	851 (2.1)	
*Any Medicaid*	14,770 (3.0)	5,521 (1.6)	3,809 (5.4)	1,893 (8.4)	3,547 (8.8)	
*Insured*	322,916 (66.3)	238,642 (67.6)	46,172 (65)	13,721 (60.9)	24,381 (60.3)	
*Unknown*	144,434 (29.7)	106,499 (30.2)	19,539 (27.5)	6,713 (29.8)	11,683 (28.9)	
**T stage**						<0.001
*Tx*	129 (0.0)	84 (0.0)	15 (0.0)	12 (0.1)	18 (0.0)	
*T0*	5 (0.0)	3 (0.0)	1 (0.0)	0 (0.0)	1 (0.0)	
*T1*	191,775 (39.4)	133,264 (37.8)	33,835 (47.7)	9,254 (41.1)	15,422 (38.1)	
*T2*	248,490 (51.0)	185,184 (52.5)	31,546 (44.4)	10,841 (48.1)	20,919 (51.7)	
*T3*	41,830 (8.6)	31,110 (8.8)	4,924 (6.9)	2,166 (9.6)	3,630 (9.0)	
*T4*	4,636 (1.0)	3241 (0.9)	662 (0.9)	261 (1.2)	472 (1.2)	
**N stage**						<0.001
*Nx*	5,659 (1.2)	3,960 (1.1)	778 (1.1)	335 (1.5)	586 (1.4)	
*N0*	474,280 (97.4)	343,954 (97.5)	69,250 (97.6)	21,890 (97.1)	39,186 (96.8)	
*N1*	6,926 (1.4)	4972 (1.4)	955 (1.3)	309 (1.4)	690 (1.7)	
**M stage**						<0.001
*M0*	486,865 (100)	352,886 (100)	70,983 (100)	22,534 (100)	40,462 (100)	
**SES composite score**						<0.001
*1*	100,029 (20.5)	78,132 (22.1)	8,122 (11.4)	8,491 (37.7)	5,284 (13.1)	
*2*	99,427 (20.4)	78,701 (22.3)	9,481 (13.4)	5,201 (23.1)	6,044 (14.9)	
*3*	93,504 (19.2)	74,423 (21.1)	11,259 (15.9)	2,475 (11.0)	5,347 (13.2)	
*4*	109,772 (22.5)	66,596 (18.9)	20,776 (29.3)	5,283 (23.4)	17,117 (42.3)	
*5*	84,005 (17.3)	54,947 (15.6)	21,340 (30.1)	1,083 (4.8)	6,635 (16.4)	
*Unknown*	128 (0.0)	87 (0.0)	5 (0.0)	1 (0.0)	35 (0.1)	
**Stage summary**						<0.001
*Localized*	423,231 (86.9)	306,113 (86.7)	63,134 (88.9)	19,255 (85.4)	34,729 (85.8)	
*Regional*	63,634 (13.1)	46,773 (13.3)	7,849 (11.1)	3,279 (14.6)	5,733 (14.2)	
**Residence type**						<0.001
*Rural*	6,316 (1.3)	5,691 (1.6)	537 (0.8)	4 (0.0)	84 (0.2)	
*Urban*	480,421 (98.7)	347,108 (98.4)	70,441 (99.2)	22,529 (100)	40,343 (99.7)	
*Unknown*	128 (0.0)	87 (0.0)	5 (0.0)	1 (0.0)	35 (0.1)	
**Treatment type**						<0.001
*None*	106,567 (21.9)	74,650 (21.2)	17,597 (24.8)	4,993 (22.2)	9,327 (23.1)	
*RT only*	162,646 (33.4)	115503 (32.7)	26,613 (37.5)	7,960 (35.3)	12,570 (31.1)	
*Surgery only*	194,237 (39.9)	145,939 (41.4)	23,297 (32.8)	8,358 (37.1)	16,643 (41.1)	
*Surgery + RT*	13,230 (2.7)	9,405 (2.7)	1,837 (2.6)	763 (3.4)	1,225 (3.0)	
*Surgery + Unknown RT*	2,681 (0.6)	2,098 (0.6)	324 (0.5)	107 (0.5)	152 (0.4)	
*Unknown*	7,249 (1.5)	5,093 (1.4)	1,272 (1.8)	349 (1.5)	535 (1.3)	
*Unknown Surgery + RT*	255 (0.1)	198 (0.1)	43 (0.1)	4 (0.0)	10 (0.0)	
**Grade^b^**						
*Well differentiated*	4,926 (1.0)	3,510 (1.0)	626 (0.9)	229 (1.0)	561 (1.4)	<0.001
*Moderately differentiated*	216,986 (44.6)	159,535 (45.2)	29,704 (41.8)	8,969 (39.8)	18,778 (46.4)	
*Poorly differentiated*	252,492 (51.9)	181,012 (51.3)	38,726 (54.6)	12,732 (56.5)	20,022 (49.5)	
*Undifferentiated*	1,044 (0.2)	732 (0.2)	181 (0.3)	46 (0.2)	85 (0.2)	
*Unknown*	11,417 (2.3)	8097 (2.3)	1,746 (2.5)	558 (2.5)	1,016 (2.5)	

Abbreviations: AAPI = Asian American/Pacific Islander; SES = socioeconomic status; RT = radiotherapy.

P-values were obtained from Kruskal-Wallis test for continuous variable and Chi-square test for categorical variables.

^a^Represents all Hispanic/Latino individuals from the study, including those reported as Hispanic/Latino, NOS.

^b^Well differentiated, moderately differentiated, and poorly differentiated histologic grading correlate with Gleason scores 2-4, 5-6, and 7-10, respectively.

**Table 2:** Patient, tumor, and treatment characteristics divided by racial and/or ethnic subgroup

*Variable*	*All*	*Non-Hispanic White*	*Mexican*	*Cuban*	*Puerto Rican*	*Dominican Republic*	*South or Central American*	*p-value*
	*N (%)*	*N (%)*	*N (%)*	*N (%)*	*N (%)*	*N (%)*	*N (%)*	
**Total patients**	366,356	352,886	8,077	788	1,284	300	3,021	
**Age at diagnosis**	66.4 ± 9.2	66.5 ± 9.2	65.9 ± 9.0	69.8 ± 8.9	65.8 ± 9.2	64.6 ± 9.0	65.0 ± 8.9	<0.001
**Marital status**								<0.001
*Married*	257,161 (70.2)	247,543 (70.1)	5,971 (73.9)	531 (67.4)	761 (59.3)	196 (65.3)	2,159 (71.5)	
*Others*	69,078 (18.9)	66,177 (18.8)	1,534 (19.0)	209 (26.5)	421 (32.8)	71 (23.7)	666 (22.0)	
*Unknown*	40,117 (11.0)	39,166 (11.1)	572 (7.1)	48 (6.1)	102 (7.9)	33 (11.0)	196 (6.5)	
**Insurance status**								<0.001
*Uninsured*	2,656 (0.7)	2,224 (0.6)	234 (2.9)	8 (1.0)	29 (2.3)	14 (4.7)	147 (4.9)	
*Any Medicaid*	7,404 (2.0)	5,521 (1.6)	1,305 (16.2)	65 (8.2)	117 (9.1)	43 (14.3)	353 (11.7)	
*Insured*	245,597 (67.0)	238,642 (67.6)	4,069 (50.4)	423 (53.7)	712 (55.5)	174 (58.0)	1,577 (52.2)	
*Unknown*	110,699 (30.2)	106,499 (30.2)	2,469 (30.6)	292 (37.1)	426 (33.2)	69 (23.0)	944 (31.2)	
**T stage**								<0.001
*Tx*	90 (0.0)	84 (0.0)	4 (0.0)	2 (0.3)	0 (0.0)	0 (0.0)	0 (0.0)	
*T0*	3 (0.0)	3 (0.0)	0 (0.0)	0 (0.0)	0 (0.0)	0 (0.0)	0 (0.0)	
*T1*	138,398 (37.8)	133,264 (37.8)	2,873 (35.6)	368 (46.7)	576 (44.9)	155 (51.7)	1,162 (38.5)	
*T2*	192,010 (52.4)	185,184 (52.5)	4,244 (52.5)	356 (45.2)	570 (44.4)	124 (41.3)	1,532 (50.7)	
*T3*	32,436 (8.9)	31,110 (8.8)	836 (10.4)	52 (6.6)	122 (9.5)	17 (5.7)	299 (9.9)	
*T4*	3,419 (0.9)	3,241 (0.9)	120 (1.5)	10 (1.3)	16 (1.2)	4 (1.3)	28 (0.9)	
**N stage**								<0.001
*Nx*	4,194 (1.1)	3,960 (1.1)	124 (1.5)	22 (2.8)	25 (1.9)	12 (4)	51 (1.7)	
*N0*	356,898 (97.4)	343,954 (97.5)	7,763 (96.1)	755 (95.8)	1,240 (96.6)	281 (93.7)	2,905 (96.2)	
*N1*	5,264 (1.4)	4,972 (1.4)	190 (2.4)	11 (1.4)	19 (1.5)	7 (2.3)	65 (2.2)	
**M stage**								<0.001
*M0*	366,356 (100)	352,886 (100)	8,077 (100)	788 (100)	1,284 (100)	300 (100)	3,021 (100)	
**SES composite score**								<0.001
*1*	79,948 (21.8)	78,132 (22.1)	731 (9.1)	117 (14.8)	366 (28.5)	77 (25.7)	525 (17.4)	
*2*	80,492 (22.0)	78,701 (22.3)	826 (10.2)	134 (17.0)	299 (23.3)	59 (19.7)	473 (15.7)	
*3*	76,149 (20.8)	74,423 (21.1)	1,153 (14.3)	58 (7.4)	205 (16.0)	43 (14.3)	267 (8.8)	
*4*	73,218 (20.0)	66,596 (18.9)	4052 (50.2)	462 (58.6)	315 (24.5)	112 (37.3)	1,681 (55.6)	
*5*	56,460 (15.4)	54,947 (15.6)	1,315 (16.3)	16 (2.0)	98 (7.6)	9 (3.0)	75 (2.5)	
*Unknown*	89 (0.0)	87 (0.0)	0 (0.0)	1 (0.1)	1 (0.1)	0 (0.0)	0 (0.0)	
**Stage summary**								<0.001
*Localized*	317,454 (86.7)	306,113 (86.7)	6,691 (82.8)	703 (89.2)	1,103 (85.9)	271 (90.3)	2,573 (85.2)	
*Regional*	48,902 (13.3)	46,773 (13.3)	1,386 (17.2)	85 (10.8)	181 (14.1)	29 (9.7)	448 (14.8)	
**Residence type**								<0.001
*Rural*	5,704 (1.6)	5,691 (1.6)	12 (0.1)	0 (0.0)	1 (0.1)	0 (0.0)	0 (0.0)	
*Urban*	360,563 (98.4)	347,108 (98.4)	8,065 (99.9)	787 (99.9)	1,282 (99.8)	300 (100)	3,021 (100)	
*Unknown*	89 (0.0)	87 (0.0)	0 (0.0)	1 (0.1)	1 (0.1)	0 (0.0)	0 (0.0)	
**Treatment type**								<0.001
*None*	77,324 (21.1)	74,650 (21.2)	1,680 (20.8)	144 (18.3)	227 (17.7)	48 (16.0)	575 (19.0)	
*RT only*	119,944 (32.7)	115,503 (32.7)	2,439 (30.2)	366 (46.4)	553 (43.1)	132 (44.0)	951 (31.5)	
*Surgery only*	151,569 (41.4)	145,939 (41.4)	3,544 (43.9)	238 (30.2)	429 (33.4)	96 (32.0)	1,323 (43.8)	
*Surgery + RT*	9,898 (2.7)	9,405 (2.7)	293 (3.6)	25 (3.2)	49 (3.8)	11 (3.7)	115 (3.8)	
*Surgery + Unknown RT*	2,154 (0.6)	2,098 (0.6)	33 (0.4)	3 (0.4)	4 (0.3)	2 (0.7)	14 (0.5)	
*Unknown*	5,264 (1.4)	5,093 (1.4)	86 (1.1)	12 (1.5)	21 (1.6)	10 (3.3)	42 (1.4)	
*Unknown Surgery + RT*	203 (0.1)	198 (0.1)	2 (0.0)	0 (0.0)	1 (0.1)	1 (0.3)	1 (0.0)	
**Grade^a^**								<0.001
*Well differentiated*	3,720 (1.0)	3,510 (1.0)	118 (1.5)	17 (2.2)	25 (1.9)	4 (1.3)	46 (1.5)	
*Moderately differentiated*	165,589 (45.2)	159,535 (45.2)	3,524 (43.6)	359 (45.6)	603 (47.0)	147 (49.0)	1,421 (47.0)	
*Poorly differentiated*	187,875 (51.3)	181,012 (51.3)	4,247 (52.6)	387 (49.1)	618 (48.1)	137 (45.7)	1,474 (48.8)	
*Undifferentiated*	755 (0.2)	732 (0.2)	12 (0.1)	2 (0.3)	3 (0.2)	1 (0.3)	5 (0.2)	
*Unknown*	8,417 (2.3)	8,097 (2.3)	176 (2.2)	23 (2.9)	35 (2.7)	11 (3.7)	75 (2.5)	

Abbreviations: SES = socioeconomic status; RT = radiotherapy.

P-values were obtained from Kruskal-Wallis test for continuous variable and Chi-square test for categorical variables.

^a^Well differentiated, moderately differentiated, and poorly differentiated histologic grading correlate with Gleason scores 2-4, 5-6, and 7-10, respectively.

**Figure 1:**
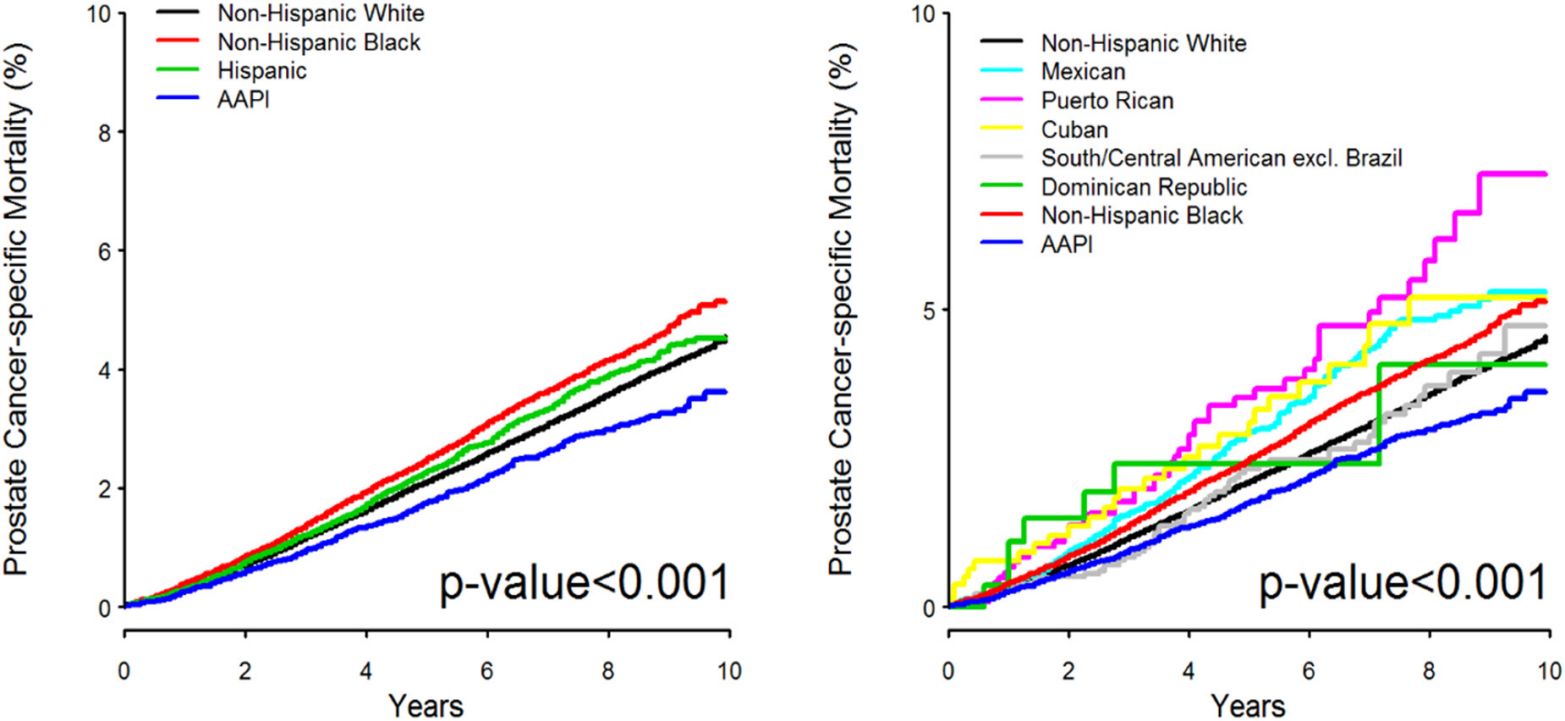
Cumulative incidence of prostate cancer-specific mortality in years divided by race and/or ethnicity: (left) Non-Hispanic White (black), Non-Hispanic Black (red), Hispanic/Latino (green), and Asian American/Pacific Islander (blue); (right) Non-Hispanic White (black), non-Hispanic Black (red), Asian American/Pacific Islander (blue), Mexican American (cyan), Puerto Rican (purple), Cuban (yellow), South or Central American excluding Brazil (grey), and Dominican Republic (green).

Figure 1 shows the cumulative incidence of PCSM among NHW, NHB, Hispanic/Latino, and AAPI men with a median follow-up of 50.4 months. NHB men have a higher PCSM than the Hispanic/Latino and NHW groups. Table 3 displays risk for PCSM in univariable analysis (UVA) and multivariable analysis (MVA) with NHW as the reference. In the UVA, NHB (HR= 1.18, 95% CI 1.12 to 1.24, *p* <0.001) men had significantly higher PCSM than NHW men; Hispanic/Latino (HR= 1.07, 95% CI 1.00 to 1.15, *p=* 0.055) men did not show statistical significance and AAPI (HR= 0.83, 95% CI 0.75 to 0.92, *p* <0.001) men trended toward lower PCSM. This was reflected in the MVA with NHW men as the reference and adjusting for age at diagnosis, disease stage summary, grade, treatment type, SES composite score, insurance status, marital status, and residence type. NHB (HR= 1.15, 95% CI 1.09 to 1.22, *p* <0.001) men had significantly worse PCSM and AAPI (HR=0.74, 95% CI 0.67 to 0.82, *p <* 0.001) men had improved PCSM, while Hispanic/Latino (HR= 1.02, 95% CI 0.95 to 1.10, *p=* 0.534) men showed no statistical significance.

**Table 3:** Fine-gray competing risks regression analyses for PCSM by race and/or ethnicity using Non-Hispanic White cohort as the reference

*Variable*	Univariable Analyses		Multivariable Analyses^a^	
	HR (95% CI)	*p-value*	HR (95% CI)	*p-value*
*Non-Hispanic White*	1.0 (reference)	-	1.0 (reference)	-
*Non-Hispanic Black*	1.18 (1.12, 1.24)	<0.001	1.15 (1.09, 1.22)	<0.001
*Hispanic/Latino*^*b*^	1.07 (1.00, 1.15)	0.055	1.02 (0.95, 1.10)	0.534
*AAPI*	0.83 (0.75, 0.92)	<0.001	0.74 (0.67, 0.82)	<0.001
*Non-Hispanic White*	1.0 (reference)	-	1.0 (reference)	-
*Mexican*	1.34 (1.17, 1.53)	<0.001	1.21 (1.05, 1.39)	0.008
*Puerto Rican*	1.70 (1.28, 2.25)	<0.001	1.71 (1.29, 2.26)	<0.001
*Cuban*	1.45 (0.99, 2.12)	0.054	1.21 (0.83, 1.78)	0.328
*South or Central American*	0.99 (0.77, 1.28)	0.957	1.11 (0.86, 1.43)	0.437
*Dominican Republic*	1.23 (0.58, 2.59)	0.586	1.60 (0.77, 3.33)	0.211

Abbreviations: HR = hazard ratio; AAPI = Asian American/Pacific Islander.

HR and p-value were obtained using Fine-Gray competing risks regression model.

^a^Multivariable analysis was conducted with competing risks regression analysis adjusting for age at diagnosis, disease stage summary, grade, treatment type, SES composite score, insurance status, marital status, and residence type.

^b^Represents all Hispanic/Latino individuals from the study, including those reported as Hispanic/Latino, NOS.

Disaggregation of the Hispanic/Latino cohort into ethnic subgroups is shown in Figure 1. Puerto Rican, Mexican, and Cuban groups had higher PCSM than not only NHW and AAPI men, but NHB men as well. The UVAs with NHW as the reference are displayed in Table 3, showing that Mexican (HR= 1.34, 95% CI 1.17 to 1.53, *p* <0.001) and Puerto Rican (HR= 1.70, 95% CI 1.28 to 2.25, *p* <0.001) men had significantly higher PCSM. In the MVA with NHW as the reference, Mexican (HR= 1.21, 95% CI 1.05 to 1.39, *p=* 0.008) and Puerto Rican (HR= 1.71, 95% CI 1.29 to 2.26, *p* <0.001) men had significantly higher PCSM compared to the NHW cohort.

In order to directly compare Hispanic/Latino subgroups at high risk for PCSM with NHB men, the most at-risk population identified thus far, we conducted additional regression models with NHB men as a reference. In the UVAs (Table 4), Puerto Rican (HR= 1.45, 95% CI 1.09 to 1.92, *p=* 0.011) men had significantly higher PCSM compared to NHB men and Mexican (HR= 1.14, 95% CI 0.99 to 1.31, *p=* 0.065) men showed no significance. In the MVA, Puerto Rican (HR= 1.50, 95% CI 1.12 to 1.96, *p*= 0.006) men continued to have significantly higher PCSM compared to NHB men, indicating that this group is at greater risk for PCSM. Mexican (HR= 1.08, 95% CI 0.93 to 1.25, *p=* 0.307) men did not show significantly different risk compared to NHB men.

**Table 4:** Fine-gray competing risks regression analyses for PCSM by race and/or ethnicity using Non-Hispanic Black cohort as the reference

*Variable*	Univariable Analyses		Multivariable Analyses^a^	
	HR (95% CI)	*p-value*	HR (95% CI)	*p-value*
*Non-Hispanic Black*	1.0 (reference)	-	1.0 (reference)	-
*Non-Hispanic White*	0.85 (0.81, 0.90)	<0.001	0.87 (0.82, 0.92)	<0.001
*Hispanic/Latino*^*b*^	0.91 (0.84, 0.99)	0.022	0.89 (0.82, 0.96)	0.005
*AAPI*	0.71 (0.63, 0.79)	<0.001	0.64 (0.57, 0.72)	<0.001
*Non-Hispanic Black*	1.0 (reference)	-	1.0 (reference)	-
*Mexican*	1.14 (0.99, 1.31)	0.065	1.08 (0.93, 1.25)	0.307
*Puerto Rican*	1.45 (1.09, 1.92)	0.011	1.50 (1.12., 1.96)	0.006
*Cuban*	1.24 (0.85, 1.81)	0.274	1.13 (0.74, 1.60)	0.679
*South or Central American*	0.84 (0.66, 1.09)	0.189	0.93 (0.73, 1.23)	0.676
*Dominican Republic*	1.04 (0.49, 2.20)	0.915	1.18 (0.64, 2.79)	0.436

Abbreviations: HR = hazard ratio; AAPI = Asian American/Pacific Islander.

HR and p-value were obtained using Fine-Gray competing risks regression model.

^a^Multivariable analysis was conducted with competing risks regression analysis adjusting for age at diagnosis, disease stage summary, grade, treatment type, SES composite score, insurance status, marital status, and residence type.

^b^Represents all Hispanic/Latino individuals from the study, including those reported as Hispanic/Latino, NOS.

## DISCUSSION

Historically, the identification of Hispanic/Latino individuals has primarily been based on political and geographic concepts [10]. Within this group, are individuals varying by language, ancestral mixture, and cultural practices [22]. Due to the unique bond shared amongst these populations and for other sociopolitical reasons, these groups have historically been aggregated. As thoughtfully outlined by Kaplan et al [7], three challenges exist when writing about race and/or ethnicity: 1) accounting for limitations of data, 2) distinguishing between race and/or ethnicity as a risk factor or as a risk marker, and 3) writing about race and/or ethnicity in a way that does not stigmatize nor create a we/they dichotomy between health professionals and minority populations. For this reason, the American Medical Association has recommended to use more descriptive terms for Hispanic/Latino individuals (e.g. Mexican American, Puerto Rican, or Cuban American), when possible [11, 23]. However, our use of the term ethnic heterogeneity is not intended to discredit the use of ethnicity or its use in identifying populations not receiving medical services, but instead to create a broader understanding of sociocultural labels and the significant clinical implications they may have on our patients.

To our knowledge, this is the first report using a national database to explore ethnic heterogeneity of Hispanic/Latino men in PCa outcomes. One Surveillance, Epidemiology, and End Results (SEER)-Medicare study included Hispanic/Latino men, but showed improved PCa related outcomes compared to NHW and NHB patients [15]. A limitation of this study was that they had a relatively small Hispanic/Latino cohort that was 66 years or older, which may not be generalizable to younger men. Two all site cancer studies that included Hispanic/Latino subgroups were conducted with state of Florida cohorts [20, 21], where percent distribution of PCa incidence is predominantly Cuban (39%), with fewer Puerto Rican (7%), Mexican (2%), South and Central American (7%), other Hispanic/Latino (10%), and Hispanic/Latino, NOS (35%) men [24]. While these state-specific demographics would be difficult to generalize nationally, the SEER database is more representative of the national picture of racial and ethnic diversity. In a recent review article, Stern et al [25] discuss reports of lower PCa incidence rates for Mexican Americans and similar or slightly higher rates for Cuban Americans and Puerto Ricans compared to NHW men [20, 26, 27]. Additionally, they discuss several other reports demonstrating the influence of SES deprivation [28], U.S. vs. foreign-born status [16], and living in predominantly Hispanic/Latino enclaves [16, 29] on mortality rates. By distinguishing between Hispanic/Latino subgroups in national populations, we are better poised to understand observed variability in disease outcomes and to address associated risk factors and risk conditions in order to assist clinical and public health decision-making.

Our findings recognize two population subgroups within the Hispanic/Latino community at similar or greater risk for PCSM than NHB men. Adjusting for the aforementioned covariates, Puerto Rican men had significantly higher PCSM compared to NHW and NHB groups. Mexican men had significantly higher PCSM compared to NHW men and showed no difference compared to NHB men. In the largest U.S. ethnic group, Mexicans comprise the majority with about 35 million people and Puerto Ricans are the second largest group with about 5 million people living in the mainland [12]. In regards to PCa disparities, an emphasis has been placed on NHB men due to higher rates of adverse outcomes compared to NHW men. The discovery that Puerto Rican men have a greater risk and Mexican American men have similar risk for PCSM compared to NHB men calls for a deeper understanding of the etiology of these disparities. Likely due to power limitations, we are unable to draw conclusions regarding Cuban and Dominican men who did not show statistical significance in regression models of PCSM.

The high rate of PCSM mortality in NHB men has been attributed to both socioeconomic [30] and biological [31] factors, a better understanding of which will contribute to improving outcomes. Efforts made to understand these factors have resulted in proposals for race-based risk classifiers when counseling patients on management strategies [32]. Although the United States Preventative Services Task Force recommends individualized decision-making about PSA screening after discussion of pros and cons with a clinician, they do recognize the higher rates of aggressive cancer in NHB men [33]. The American Cancer Society recommends that all men with average risk and older than 50 years should receive PSA screening information to make an informed decision with their provider about being tested. They recommend this information be offered to NHB men and those with a family history of PCa at 45 years of age and men with more than one first-degree relative who had PCa at an early age should be offered such information at 40 years of age [34]. Variations in screening guideline recommendations are a reflection of the impact made by the identification and further characterization of these at-risk men. Although PSA is a suboptimal screening tool that will likely be enhanced/substituted over time (i.e. PCA3, PHI, 4K score), our findings indicate that consideration be given to the ethnic background of Hispanic/Latino men.

A strength of our study is the use of a contemporary population-based cancer registry containing a large, diverse cohort. However, the SEER database has potential limitations. By using the NHIA recode for ethnicity, there is potential for U.S. born Hispanic/Latino men to have missing country of origin or ancestry data due to lack of affiliation with Hispanic/Latino identity or no specific information is provided [35]. For this reason, U.S. born Hispanic/Latino men identified by surname only are thus labeled as Hispanic/Latino, NOS. In our cohort, less than 50% of the Hispanic/Latino men were identified to belong to a specific Hispanic/Latino subgroup, which could have weakened the power of subgroup analyses. The inability to further specify the region of origin or ancestry within the South or Central American group was also a source of limitation due to the potential for within-group differences. Though Cuban, South or Central American, and Dominican subgroups were not found to be significantly different than NHW or NHB men, the cumulative incidence plots (Figure 1B) suggest diversity in these populations requiring further investigation. Beginning with the 2014 submission, SEER no longer provides birthplace data due to disproportional documentation rates in living vs. deceased cases [36–38]. With immigrants constituting a significant portion of the Hispanic/Latino community, the absence of this information limits our ability to account for potential differences between U.S. and foreign-born Hispanic/Latino men.

Another potential limitation in our study is the use of poverty, education, and residence type information collected at the county level, not the individual or census tract level. While several publications have described the benefits of monitoring inequalities through census tract level data [39], this information is not available in SEER. PSA levels were not made available on the 2015 SEER data release due to questionable accuracy, thus not included in this analysis [19]. According to guidelines from the National Comprehensive Cancer Network, patients diagnosed with metastatic PCa are primarily treated with chemotherapy and/or hormone therapy, but SEER does not provide such data. For this reason, patients with metastatic disease were excluded to eliminate these potential confounders when adjusting for treatment type in the MVA. Lastly, a comorbidity index is only available in the SEER-Medicare linked data. We chose to use the entire SEER dataset to maximize the power in the examination of Hispanic/Latino subgroups. The use of cancer-specific mortality as an endpoint minimizes the influence of comorbidity as a confounder. The findings that such disparities in PCSM exist should be recognized, regardless of potential influences.

In summary, our data indicate that ethnic heterogeneity amongst Hispanic/Latino populations in the United States significantly contributes to PCSM, which should be considered in guidelines for clinical practice. Two distinct subgroups had PCSM rates that were higher than all other racial and/or ethnic groups when compared to NHW men. Compared to NHB men, PCSM was significantly worse in Puerto Rican men and showed no difference in Mexican men. As appreciated in the NHB population, this type of information drives screening policy recommendations and affects management recommendations, including active surveillance. Further investigation into potentially contributing biological, socioeconomic, and/or sociocultural factors unique to certain Hispanic/Latino subgroups is warranted.

## MATERIALS AND METHODS

PCa incidence and outcome data was obtained from 18 registries of the November 2015 submission offered by the SEER program of the National Cancer Institute, which includes about 30% of the U.S. population. Analyses of these data are considered nonhuman subjects research by the U.S. Department of Health and Human Services’ Office for Human Research Protection because it is de-identified and publicly available, which does not require institutional review board approval.

### Definitions

Since race and ethnicity are constantly evolving concepts and used ubiquitously in medical literature [40], the terms are defined accordingly. Race is a category of humankind that shares certain distinctive physical traits [41]. Ethnicity refers to groups of people classed according to common racial, national, tribal, religious, linguistic, or cultural origin or background [41]. According to the U.S. Census Bureau, Hispanic or Latino refers to those who identify with being Mexican, Puerto Rican, Cuban, Dominican, Central or South American, or from a country of Spanish-speaking origin [42]. However, others have stated that the term Latino includes any person with origins from Latin America, including Brazil [10]. Since the SEER database does not include Brazilian Americans in their Hispanic origin recode, we use Hispanic/Latino to denote all U.S. persons whose origins can be traced to the Spanish-speaking regions of Latin America.

### National hispanic identification algorithm

From the SEER database, we used the National Hispanic Identification Algorithm (NHIA) origin recode designed by the North American Association of Central Cancer Registries (NAACCR) for ethnicity data [43]. This algorithm evaluates surname, race, ethnicity self-identification, and birthplace to assign a code of non-Hispanic, a specific Hispanic/Latino subgroup, or Hispanic/Latino, not otherwise specified (NOS). Only those who self-identify or have heavily Hispanic surnames and are not of AAPI racial background are concluded to have Hispanic/Latino ethnicity. The successful use of this recode has been exhibited in similar publications [44].

### Patient population

Men diagnosed with PCa from 2000-2013 (n= 791,234) were extracted from the SEER database. All men with Mx/M1 staging or distant disease (n= 285,897) were excluded to avoid any confounding variables related to chemotherapy or hormone therapy, which are not documented in the SEER program. We identified 505,337 men with local-regional PCa from 2000-2013 (Supplementary Figure 1). Our final cohort (n= 486,865) compared the following racial and/or ethnic groups: NHW (n= 352,886), NHB (n= 70,983), Hispanic/Latino (n= 40,462), and AAPI (n= 22,534). Other racial/ethnic groups (n= 18,472) were not included in the analysis. Additionally, we compared Hispanic/Latino subgroups: Mexican (n= 8,077), Puerto Rican (n= 1,284), South or Central American (n= 3,021), Cuban (n= 788), and Dominican (n= 300). Any patient identified as Hispanic/Latino, NOS (n= 26,992) without specific country of origin or ancestry information was excluded from the Hispanic/Latino subgroup analysis.

### Study variables

Variables that were evaluated include race and/or ethnicity (NHW, NHB, Hispanic/Latino, or AAPI), ethnic subgroups (Mexican, Puerto Rican, Cuban, Dominican, or South or Central American excluding Brazil), age at diagnosis, TNM staging, disease stage summary (localized vs. regional disease), grade, treatment type (none vs. surgery only vs. radiation treatment [RT] only vs. surgery + RT vs. surgery + unknown RT vs. unknown surgery + RT vs. unknown), SES composite score, insurance status (uninsured vs. any Medicaid vs. insured vs. unknown), marital status (married vs. others vs. unknown), and residence type (rural vs. urban).

### Socioeconomic status composite score

We collected three county level variables representative of SES: 1) education: percent of adults at age 25 years or older with less than a 12^th^ grade education; 2) poverty: percent of people living below the poverty line; and 3) income: median annual household income. Each individual variable was standardized into values ranging from 0 to 1 or low SES to high SES, respectively. All three variables were then added and the sum for each case was equally weighted to create an SES composite score based on previously published methodologies to provide a multidimensional standardized measure of socioeconomic conditions [45].

### Statistical analyses

The cumulative incidence of PCSM for all racial and/or ethnic groups was examined generally (Figure 1) and with categorical age groups: <65 years old (Supplementary Figure 2) and ≥65 years old (Supplementary Figure 3). In the supplement, patient characteristic tables were provided for men <65 years old (Supplementary Tables 1 and 2) and ≥65 years old (Supplementary Tables 3 and 4). Five-year cumulative incidence rates for PCSM, non-PCSM, and all-cause mortality are reported in Supplementary Table 5. We used Fine-Gray competing risks regression for the UVA and MVA [46]. In the MVA, we included age at diagnosis, disease stage summary, grade, treatment type, SES composite score, insurance status, marital status, and residence type for adjustment. In separate UVA and MVA, NHW and NHB were used as reference groups. Median follow-up among surviving patients was 50.4 months (0-119 months). All statistical tests were two-sided, and p-values less than 0.05 were considered statistically significant. Analyses involving competing risks were performed using R software (http://www.r-project.org) and all other analyses were carried out using SAS (SAS Institute, Cary, NC).

## SUPPLEMENTARY MATERIALS FIGURES AND TABLES





## Abbreviations

PCa: prostate cancer
PCSM: prostate cancer specific mortality
NHW: Non-Hispanic White
NHB: Non-Hispanic Black
AAPI: Asian American/Pacific Islander
UVA: univariable analysis
MVA: multivariable analysis
SES: socioeconomic status
SEER: Surveillance, Epidemiology, and End Results
NHIA: National Hispanic Identification Algorithm
NAACCR: North American Association of Central Cancer Registries
NOS: not otherwise specified
