# Supplementary material for: Ethnic heterogeneity and prostate cancer mortality in Hispanic/Latino men: a population-based study
Source: Oncotarget. 2017 Jul 6;8(41):69709–21. doi: 10.18632/oncotarget.19068 (PMC5642510; doi:10.18632/oncotarget.19068)
Supplement: Supplementary file 1 [file oncotarget-08-69709-s001.pdf]

# Ethnic heterogeneity and prostate cancer mortality in Hispanic/ Latino men: a population-based study

## SUPPLEMENTARY FIGURES AND TABLES

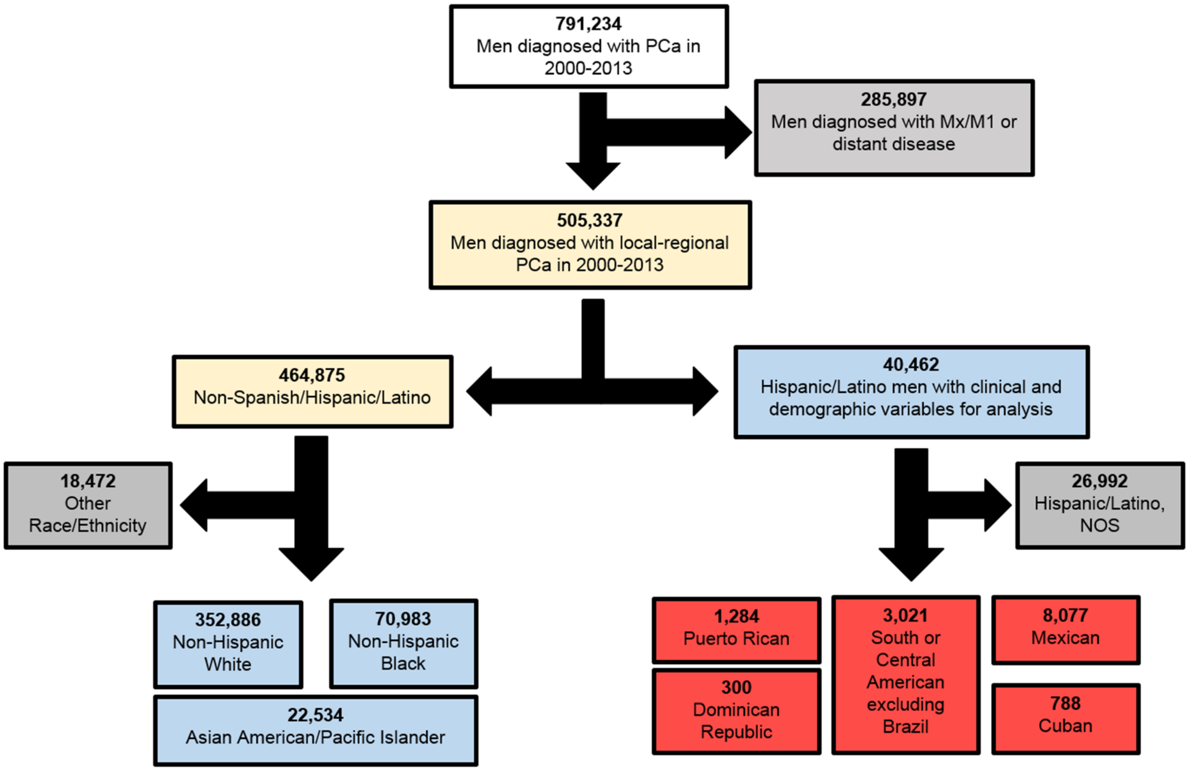


**Supplementary Figure 1:** Flowchart displaying initial SEER-derived cohort (white), excluded patients (grey), local-regional prostate cancer cases (yellow), included racial/ethnic groups (blue), and Hispanic/Latino subgroups (red).

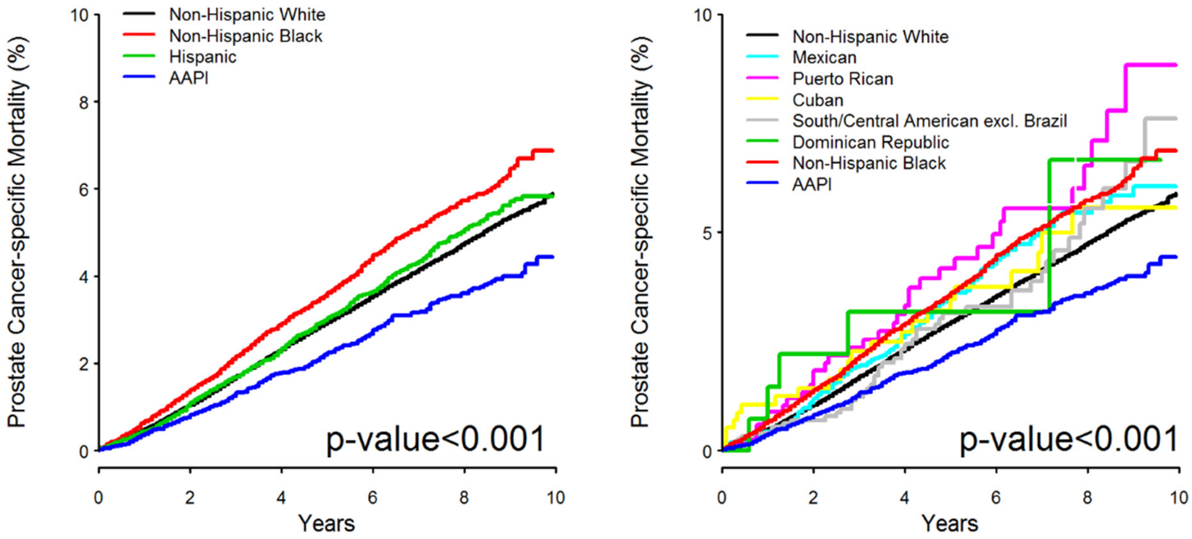


**Supplementary Figure 2:** Cumulative incidence of prostate cancer-specific mortality in years for men <65 years old divided by (left) racial/ethnic groups: Non-Hispanic White (black), Non-Hispanic Black (red), Hispanic/Latino (green), Asian American/Pacific Islander (blue) and (right) racial/ethnicsubgroups: Non-Hispanic White (black), Non-Hispanic Black (red), Asian American/Pacific Islander (blue), Mexican American (cyan), Puerto Rican (purple), Cuban (yellow), South or Central American excluding Brazil (grey), and Dominican Republic (green).

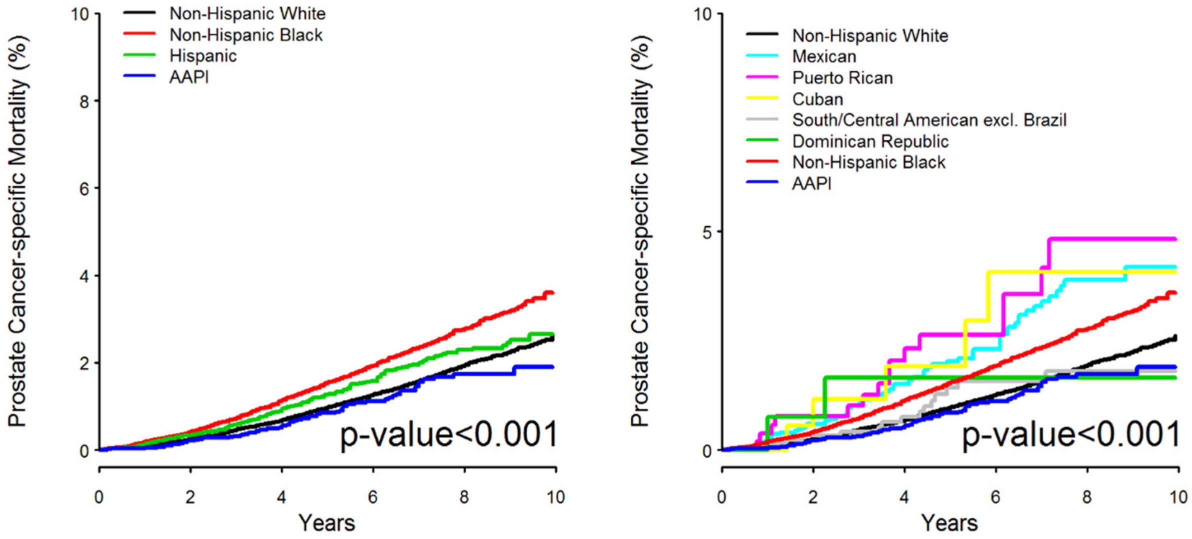


**Supplementary Figure 3:** Cumulative incidence of prostate cancer-specific mortality in years for men ≥65 years old divided by (left) racial/ethnic groups: Non-Hispanic White (black), Non-Hispanic Black (red), Hispanic/Latino (green), Asian American/Pacific Islander (blue) and (right) racial/ethnicsubgroups: Non-Hispanic White (black), Non-Hispanic Black (red), Asian American/Pacific Islander (blue), Mexican American (cyan), Puerto Rican (purple), Cuban (yellow), South or Central American excluding Brazil (grey), and Dominican Republic (green).

**Supplemental Table 1: Patient, tumor, and treatment characteristics divided by racial and/or ethnic group for men <65 years old**

See Supplementary File 1

**Supplemental Table 2: Patient, tumor, and treatment characteristics divided by racial and/or ethnic subgroup for men <65 years old**

See Supplementary File 1

**Supplemental Table 3: Patient, tumor, and treatment characteristics divided by racial and/or ethnic group for men ≥65 years old**

See Supplementary File 1

**Supplemental Table 4: Patient, tumor, and treatment characteristics divided by racial and/or ethnic subgroup for men ≥65 years old**

See Supplementary File 1

**Supplementary Table 5: Five-year prostate cancer specific mortality, non-prostate cancer-specific mortality, and all-cause mortality for racial/ethnic groups and subgroups**

| Race/ethnicity | Five-year PCSM (%) | 95% CI | Five-year non-PCSM (%) | 95% CI | Five-year ACM (%) | 95% CI |
|---|---|---|---|---|---|---|
| *Non-Hispanic White* | 2.11 | (2.06, 2.17) | 10.56 | (10.44, 10.68) | 12.67 | (12.54, 12.80) |
| *Non-Hispanic Black* | 2.50 | (2.37, 2.64) | 11.86 | (11.57, 12.15) | 14.36 | (14.05, 14.67) |
| *Hispanic/Latino*[a] | 2.29 | (2.12, 2.47) | 8.71 | (8.38, 9.05) | 11.00 | (10.63, 11.38) |
| *Asian American/ Pacific Islander* | 1.76 | (1.57, 1.98) | 9.45 | (8.99, 9.92) | 11.21 | (10.72, 11.72) |
| *Mexican* | 2.95 | (2.52, 3.43) | 10.43 | (9.63, 11.27) | 13.38 | (12.48, 14.31) |
| *Puerto Rican* | 3.52 | (2.49, 4.82) | 15.22 | (13.00, 17.60) | 18.74 | (16.31, 21.30) |
| *Cuban* | 3.11 | (1.95, 4.68) | 15.03 | (12.33, 17.98) | 18.13 | (15.19, 21.28) |
| *South or Central American* | 2.32 | (1.72, 3.06) | 6.80 | (5.76, 7.95) | 9.12 | (7.91, 10.43) |
| *Dominican* | 2.41 | (0.99, 4.94) | 8.20 | (4.75, 12.82) | 10.60 | (6.72, 15.50) |

Abbreviations: PCSM = prostate cancer specific mortality; ACM= all-cause mortality; PCa = prostate cancer; CI = confidence interval.

[a]Represents all Hispanic/Latino individuals from the study, including those reported as Hispanic/Latino, NOS.
