# Supplementary material for: Ethnic heterogeneity and prostate cancer mortality in Hispanic/Latino men: a population-based study
Source: Oncotarget. 2017 Jul 6;8(41):69709–21. doi: 10.18632/oncotarget.19068 (PMC5642510; doi:10.18632/oncotarget.19068)
Supplement: Supplementary file 2 [file oncotarget-08-69709-s002.docx]

| **Supplemental Table 1. Patient, tumor, and treatment characteristics divided by racial and/or ethnic group for men <65 years old** | | | | | | |
| --- | --- | --- | --- | --- | --- | --- |
| *Variable* | *All* | *Non- Hispanic White* | *Non-Hispanic Black* | *AAPI* | *Hispanic/Latino*^a^ |  |
|  | *N (%)* | *N (%)* | *N (%)* | *N (%)* | *N (%)* | *p-value* |
| **Total patients** | 214,385 | 149,858 | 38,944 | 7,860 | 17,723 |  |
| **Age at diagnosis** | 57.7 ± 5.0 | 58 ± 4.8 | 56.8 ± 5.4 | 58.4 ± 4.7 | 57.4 ± 5.2 | <0.001 |
| **Marital status** |  | | | | | <0.001 |
| *Married* | 146,543 (68.4) | 106906 (71.3) | 21,256 (54.6) | 6,110 (77.7) | 12,271 (69.2) |  |
| *Others* | 47,290 (22.1) | 28,818 (19.2) | 13,686 (35.1) | 1,070 (13.6) | 3,716 (21) |  |
| *Unknown* | 20,552 (9.6) | 14,134 (9.4) | 4,002 (10.3) | 680 (8.7) | 1,736 (9.8) |  |
| **Insurance status** |  | | | | | <0.001 |
| *Uninsured* | 3,730 (1.7) | 1,721 (1.1) | 1,233 (3.2) | 116 (1.5) | 660 (3.7) |  |
| *Any Medicaid* | 6,138 (2.9) | 2,379 (1.6) | 2,256 (5.8) | 292 (3.7) | 1,211 (6.8) |  |
| *Insured* | 144,607 (67.5) | 102,688 (68.5) | 25,371 (65.1) | 5,395 (68.6) | 11,153 (62.9) |  |
| *Unknown* | 59,910 (27.9) | 43,070 (28.7) | 10,084 (25.9) | 2,057 (26.2) | 4,699 (26.5) |  |
| **T stage** |  | | | | | <0.001 |
| *Tx* | 37 (0.0) | 21 (0.0) | 6 (0.0) | 4 (0.1) | 6 (0.0) |  |
| *T0* | 2 (0.0) | 1 (0.0) | . (.) | . (.) | 1 (0.0) |  |
| *T1* | 64,675 (30.2) | 41,117 (27.4) | 15,890 (40.8) | 2,392 (30.4) | 5,276 (29.8) |  |
| *T2* | 124,497 (58.1) | 90,539 (60.4) | 19,288 (49.5) | 4,404 (56.0) | 10,266 (57.9) |  |
| *T3* | 22,853 (10.7) | 16,609 (11.1) | 3,327 (8.5) | 958 (12.2) | 1,959 (11.1) |  |
| *T4* | 2,321 (1.1) | 1,571 (1.0) | 433 (1.1) | 102 (1.3) | 215 (1.2) |  |
| **N stage** |  | | | | | <0.001 |
| *Nx* | 1,805 (0.8) | 1,199 (0.8) | 337 (0.9) | 72 (0.9) | 197 (1.1) |  |
| *N0* | 208,947 (97.5) | 146,153 (97.5) | 37,985 (97.5) | 7,651 (97.3) | 17,158 (96.8) |  |
| *N1* | 3,633 (1.7) | 2,506 (1.7) | 622 (1.6) | 137 (1.7) | 368 (2.1) |  |
| **M stage** |  | | | | | <0.001 |
| *M0* | 214,385 (100) | 149,858 (100) | 38,944 (100) | 7,860 (100) | 17,723 (100) |  |
| **SES composite score** |  | | | | | <0.001 |
| *1* | 45,160 (21.1) | 35,011 (23.4) | 4,658 (12.0) | 2,961 (37.7) | 2,530 (14.3) |  |
| *2* | 44,458 (20.7) | 34,501 (23.0) | 5,430 (13.9) | 1,746 (22.2) | 2,781 (15.7) |  |
| *3* | 41,467 (19.3) | 31,898 (21.3) | 6,364 (16.3) | 902 (11.5) | 2,303 (13.0) |  |
| *4* | 47,609 (22.2) | 26,889 (17.9) | 11,324 (29.1) | 1,860 (23.7) | 7,536 (42.5) |  |
| *5* | 35,634 (16.6) | 21,520 (14.4) | 11,163 (28.7) | 391 (5.0) | 2,560 (14.4) |  |
| *Unknown* | 57 (0.0) | 39 (0.0) | 5 (0.0) | . (.) | 13 (0.1) |  |
| **Stage summary** |  | | | | | <0.001 |
| *Localized* | 178,251 (83.1) | 123,891 (82.7) | 33,459 (85.9) | 6,340 (80.7) | 1,4561 (82.2) |  |
| *Regional* | 36,134 (16.9) | 25,967 (17.3) | 5,485 (14.1) | 1,520 (19.3) | 3,162 (17.8) |  |
| **Residence type** |  | | | | | <0.001 |
| *Rural* | 2,345 (1.1) | 2,062 (1.4) | 254 (0.7) | 2 (0) | 27 (0.2) |  |
| *Urban* | 211,983 (98.9) | 147,757 (98.6) | 38,685 (99.3) | 7,858 (100) | 17,683 (99.8) |  |
| *Unknown* | 57 (0) | 39 (0) | 5 (0) | . (.) | 13 (0.1) |  |
| **Treatment type** |  | | | | | <0.001 |
| *None* | 33,298 (15.5) | 21,459 (14.3) | 7,418 (19.0) | 1,270 (16.2) | 3,151 (17.8) |  |
| *RT only* | 54,028 (25.2) | 35,205 (23.5) | 12,977 (33.3) | 1,905 (24.2) | 3,941 (22.2) |  |
| *Surgery only* | 116,003 (54.1) | 85,560 (57.1) | 16,526 (42.4) | 4,180 (53.2) | 9,737 (54.9) |  |
| *Surgery + RT* | 6,879 (3.2) | 4,762 (3.2) | 1,149 (3.0) | 354 (4.5) | 614 (3.5) |  |
| *Surgery + Unknown RT* | 1,550 (0.7) | 1,190 (0.8) | 225 (0.6) | 50 (0.6) | 85 (0.5) |  |
| *Unknown* | 2,534 (1.2) | 1,621 (1.1) | 623 (1.6) | 99 (1.3) | 191 (1.1) |  |
| *Unknown Surgery + RT* | 93 (0.0) | 61 (0.0) | 26 (0.1) | 2 (0.0) | 4 (0.0) |  |
| **Grade**^b^ |  |  |  |  |  | <0.001 |
| *Well differentiated* | 2,016 (0.9) | 1,406 (0.9) | 313 (0.8) | 76 (1.0) | 221 (1.2) |  |
| *Moderately differentiated* | 103,067 (48.1) | 73,609 (49.1) | 17,108 (43.9) | 3,524 (44.8) | 8,826 (49.8) |  |
| *Poorly differentiated* | 104,874 (48.9) | 71,889 (48.0) | 20,642 (53.0) | 4,081 (51.9) | 8,262 (46.6) |  |
| *Undifferentiated* | 383 (0.2) | 238 (0.2) | 98 (0.3) | 15 (0.2) | 32 (0.2) |  |
| *Unknown* | 4,045 (1.9) | 2,716 (1.8) | 783 (2.0) | 164 (2.1) | 382 (2.2) |  |
| Abbreviations: AAPI = Asian American/Pacific Islander; SES = socioeconomic status; RT = radiotherapy  P-values were obtained from Kruskal-Wallis test for continuous variable and Chi-square test for categorical variables.  ^a^Represents all Hispanic/Latino individuals from the study, including those reported as Hispanic/Latino, NOS.  ^b^Well differentiated, moderately differentiated, and poorly differentiated histologic grading correlate with Gleason scores 2-4, 5-6, and 7-10, respectively. | | | | | | |

| **Supplemental Table 2. Patient, tumor, and treatment characteristics divided by racial and/or ethnic subgroup for men <65 years old** | | | | | | | | |
| --- | --- | --- | --- | --- | --- | --- | --- | --- |
| *Variable* | *All* | *Non-Hispanic White* | *Mexican* | *Cuban* | *Puerto Rican* | *Dominican Republic* | *South or Central American* |  |
|  | *N (%)* | *N (%)* | *N (%)* | *N (%)* | *N (%)* | *N (%)* | *N (%)* | *p-value* |
| **Total patients** | 155,620 | 149,858 | 3,394 | 207 | 562 | 151 | 1,448 |  |
| **Age at diagnosis** | 58 ± 4.8 | 58 ± 4.8 | 57.5 ± 4.1 | 58.3 ± 5.0 | 57.5 ± 5.0 | 57.4 ± 5.1 | 57.6 ± 5.1 | <0.001 |
| **Marital status** |  | | | | | | | <0.001 |
| *Married* | 111,123 (71.4) | 106,906 (71.3) | 2,598 (76.5) | 126 (60.9) | 336 (59.8) | 101 (66.9) | 1,056 (72.9) |  |
| *Others* | 30,023 (19.3) | 28,818 (19.2) | 596 (17.6) | 72 (34.8) | 190 (33.8) | 34 (22.5) | 313 (21.6) |  |
| *Unknown* | 14,474 (9.3) | 14,134 (9.4) | 200 (5.9) | 9 (4.3) | 36 (6.4) | 16 (10.6) | 79 (5.5) |  |
| **Insurance status** |  | | | | | | | <0.001 |
| *Uninsured* | 2,055 (1.3) | 1,721 (1.1) | 191 (5.6) | 5 (2.4) | 22 (3.9) | 12 (7.9) | 104 (7.2) |  |
| *Any Medicaid* | 3,001 (1.9) | 2,379 (1.6) | 427 (12.6) | 17 (8.2) | 46 (8.2) | 16 (10.6) | 116 (8.0) |  |
| *Insured* | 105,777 (68.0) | 102,688 (68.5) | 1,790 (52.7) | 116 (56.0) | 329 (58.5) | 88 (58.3) | 766 (52.9) |  |
| *Unknown* | 44,787 (28.8) | 43,070 (28.7) | 986 (29.1) | 69 (33.3) | 165 (29.4) | 35 (23.2) | 462 (31.9) |  |
| **T stage** |  | | | | | | | <0.001 |
| *Tx* | 24 (0.0) | 21 (0.0) | 2 (0.1) | 1 (0.5) | . (.) | . (.) | . (.) |  |
| *T0* | 1 (0.0) | 1 (0.0) | . (.) | . (.) | . (.) | . (.) | . (.) |  |
| *T1* | 42,832 (27.5) | 41,117 (27.4) | 965 (28.4) | 71 (34.3) | 196 (34.9) | 57 (37.7) | 426 (29.4) |  |
| *T2* | 93,803 (60.3) | 90,539 (60.4) | 1,941 (57.2) | 104 (50.2) | 293 (52.1) | 81 (53.6) | 845 (58.4) |  |
| *T3* | 17,315 (11.1) | 16,609 (11.1) | 435 (12.8) | 27 (13.0) | 68 (12.1) | 11 (7.3) | 165 (11.4) |  |
| *T4* | 1,645 (1.1) | 1571 (1.0) | 51 (1.5) | 4 (1.9) | 5 (0.9) | 2 (1.3) | 12 (0.8) |  |
| **N stage** |  | | | | | | | <0.001 |
| *Nx* | 1,277 (0.8) | 1,199 (0.8) | 47 (1.4) | 4 (1.9) | 9 (1.6) | 4 (2.6) | 14 (1) |  |
| *N0* | 151,683 (97.5) | 146,153 (97.5) | 3,249 (95.7) | 197 (95.2) | 544 (96.8) | 142 (94) | 1,398 (96.5) |  |
| *N1* | 2,660 (1.7) | 2,506 (1.7) | 98 (2.9) | 6 (2.9) | 9 (1.6) | 5 (3.3) | 36 (2.5) |  |
| **M stage** |  | | | | | | | <0.001 |
| *M0* | 155,620 (100) | 149,858 (100) | 3,394 (100) | 207 (100) | 562 (100) | 151 (100) | 1,448 (100) |  |
| **SES composite score** |  | | | | | | | <0.001 |
| *1* | 35,845 (23.0) | 35,011 (23.4) | 323 (9.5) | 43 (20.8) | 162 (28.8) | 40 (26.5) | 266 (18.4) |  |
| *2* | 35,253 (22.7) | 34,501 (23.0) | 323 (9.5) | 40 (19.3) | 149 (26.5) | 24 (15.9) | 216 (14.9) |  |
| *3* | 32,617 (21.0) | 31,898 (21.3) | 466 (13.7) | 18 (8.7) | 76 (13.5) | 25 (16.6) | 134 (9.3) |  |
| *4* | 29,745 (19.1) | 26,889 (17.9) | 1,785 (52.6) | 99 (47.8) | 124 (22.1) | 55 (36.4) | 793 (54.8) |  |
| *5* | 22,119 (14.2) | 21,520 (14.4) | 497 (14.6) | 6 (2.9) | 50 (8.9) | 7 (4.6) | 39 (2.7) |  |
| *Unknown* | 41 (0.0) | 39 (0.0) | . (.) | 1 (0.5) | 1 (0.2) | . (.) | . (.) |  |
| **Stage summary** |  | | | | | | | <0.001 |
| *Localized* | 128,522 (82.6) | 123,891 (82.7) | 2,670 (78.7) | 166 (80.2) | 463 (82.4) | 131 (86.8) | 1,201 (82.9) |  |
| *Regional* | 27,098 (17.4) | 25,967 (17.3) | 724 (21.3) | 41 (19.8) | 99 (17.6) | 20 (13.2) | 247 (17.1) |  |
| **Residence type** |  | | | | | | | <0.001 |
| *Rural* | 2,064 (1.3) | 2,062 (1.4) | 1 (0.0) | . (.) | 1 (0.2) | . (.) | . (.) |  |
| *Urban* | 153,515 (98.6) | 147,757 (98.6) | 3,393 (100) | 206 (99.5) | 560 (99.6) | 151 (100) | 1,448 (100) |  |
| *Unknown* | 41 (0.0) | 39 (0.0) | . (.) | 1 (0.5) | 1 (0.2) | . (.) | . (.) |  |
| **Treatment type** |  | | | | | | | <0.001 |
| *None* | 22,384 (14.4) | 21,459 (14.3) | 581 (17.1) | 25 (12.1) | 78 (13.9) | 16 (10.6) | 225 (15.5) |  |
| *RT only* | 36,532 (23.5) | 35,205 (23.5) | 723 (21.3) | 63 (30.4) | 175 (31.1) | 52 (34.4) | 314 (21.7) |  |
| *Surgery only* | 88,743 (57.0) | 85,560 (57.1) | 1,899 (56.0) | 109 (52.7) | 274 (48.8) | 69 (45.7) | 832 (57.5) |  |
| *Surgery + RT* | 4,997 (3.2) | 4,762 (3.2) | 141 (4.2) | 7 (3.4) | 27 (4.8) | 6 (4.0) | 54 (3.7) |  |
| *Surgery + Unknown RT* | 1,218 (0.8) | 1,190 (0.8) | 15 (0.4) | 1 (0.5) | 2 (0.4) | 1 (0.7) | 9 (0.6) |  |
| *Unknown* | 1,683 (1.1) | 1, 621 (1.1) | 35 (1.0) | 2 (1.0) | 6 (1.1) | 6 (4.0) | 13 (0.9) |  |
| *Unknown Surgery + RT* | 63 (0.0) | 61 (0.0) | . (.) | . (.) | . (.) | 1 (0.7) | 1 (0.1) |  |
| **Grade**^a^ |  |  |  |  |  |  |  | 0.001 |
| *Well differentiated* | 1,486 (1.0) | 1,406 (0.9) | 37 (1.1) | 3 (1.4) | 15 (2.7) | 3 (2.0) | 22 (1.5) |  |
| *Moderately differentiated* | 76,412 (49.1) | 73,609 (49.1) | 1,609 (47.4) | 109 (52.7) | 276 (49.1) | 80 (53.0) | 729 (50.3) |  |
| *Poorly differentiated* | 74,660 (48.0) | 71,889 (48.0) | 1,691 (49.8) | 89 (43.0) | 258 (45.9) | 62 (41.1) | 671 (46.3) |  |
| *Undifferentiated* | 244 (0.2) | 238 (0.2) | 3 (0.1) | . (.) | 1 (0.2) | 1 (0.7) | 1 (0.1) |  |
| *Unknown* | 2,818 (1.8) | 2,716 (1.8) | 54 (1.6) | 6 (2.9) | 12 (2.1) | 5 (3.3) | 25 (1.7) |  |
| Abbreviations: SES = socioeconomic status; RT = radiotherapy  P-values were obtained from Kruskal-Wallis test for continuous variable and Chi-square test for categorical variables.  ^a^Well differentiated, moderately differentiated, and poorly differentiated histologic grading correlate with Gleason scores 2-4, 5-6, and 7-10, respectively. | | | | | | | | |

| **Supplemental Table 3. Patient, tumor, and treatment characteristics divided by racial and/or ethnic group for men ≥65 years old** | | | | | | |
| --- | --- | --- | --- | --- | --- | --- |
| *Variable* | *All* | *Non- Hispanic White* | *Non-Hispanic Black* | *AAPI* | *Hispanic/Latino*^a^ |  |
|  | *N (%)* | *N (%)* | *N (%)* | *N (%)* | *N (%)* | *p-value* |
| **Total patients** | 272,480 | 203,028 | 32,039 | 14,674 | 22,739 |  |
| **Age at diagnosis** | 72.6 ± 6.0 | 72.7 ± 6.0 | 71.6 ± 5.6 | 72.3 ± 5.7 | 73 ± 6.1 | <0.001 |
| **Marital status** |  | | | | | <0.001 |
| *Married* | 183,711 (67.4) | 140,637 (69.3) | 171,88 (53.6) | 10,939 (74.5) | 149,47 (65.7) |  |
| *Others* | 54,870 (20.1) | 37,359 (18.4) | 10,606 (33.1) | 2,071 (14.1) | 4,834 (21.3) |  |
| *Unknown* | 33,899 (12.4) | 25,032 (12.3) | 4,245 (13.2) | 1,664 (11.3) | 2,958 (13) |  |
| **Insurance status** |  | | | | | <0.001 |
| *Uninsured* | 1,015 (0.4) | 503 (0.2) | 230 (0.7) | 91 (0.6) | 191 (0.8) |  |
| *Any Medicaid* | 8,632 (3.2) | 3,142 (1.5) | 1,553 (4.8) | 1,601 (10.9) | 2,336 (10.3) |  |
| *Insured* | 178,309 (65.4) | 135,954 (67) | 20,801 (64.9) | 8,326 (56.7) | 13,228 (58.2) |  |
| *Unknown* | 84,524 (31.0) | 63,429 (31.2) | 9,455 (29.5) | 4,656 (31.7) | 6,984 (30.7) |  |
| **T stage** |  | | | | | <0.001 |
| *Tx* | 92 (0.0) | 63 (0.0) | 9 (0.0) | 8 (0.1) | 12 (0.1) |  |
| *T0* | 3 (0.0) | 2 (0.0) | 1 (0.0) | . (.) | . (.) |  |
| *T1* | 127,100 (46.6) | 92,147 (45.4) | 17,945 (56) | 6,862 (46.8) | 10,146 (44.6) |  |
| *T2* | 123,993 (45.5) | 94,645 (46.6) | 12,258 (38.3) | 6,437 (43.9) | 10,653 (46.8) |  |
| *T3* | 18,977 (7.0) | 14,501 (7.1) | 1,597 (5) | 1,208 (8.2) | 1,671 (7.3) |  |
| *T4* | 2,315 (0.8) | 1,670 (0.8) | 229 (0.7) | 159 (1.1) | 257 (1.1) |  |
| **N stage** |  | | | | | <0.001 |
| *Nx* | 3,854 (1.4) | 2,761 (1.4) | 441 (1.4) | 263 (1.8) | 389 (1.7) |  |
| *N0* | 265,333 (97.4) | 197,801 (97.4) | 31,265 (97.6) | 14,239 (97.0) | 22,028 (96.9) |  |
| *N1* | 3,293 (1.2) | 2,466 (1.2) | 333 (1.0) | 172 (1.2) | 322 (1.4) |  |
| **M stage** |  | | | | | <0.001 |
| *M0* | 272,480 (100) | 203,028 (100) | 32,039 (100) | 14,674 (100) | 22,739 (100) |  |
| **SES composite score** |  | | | | | <0.001 |
| *1* | 54,869 (20.1) | 43,121 (21.2) | 3,464 (10.8) | 5,530 (37.7) | 2,754 (12.1) |  |
| *2* | 54,969 (20.2) | 44,200 (21.8) | 4,051 (12.6) | 3,455 (23.5) | 3,263 (14.3) |  |
| *3* | 52,037 (19.1) | 42,525 (20.9) | 4,895 (15.3) | 1,573 (10.7) | 3,044 (13.4) |  |
| *4* | 62,163 (22.8) | 39,707 (19.6) | 9,452 (29.5) | 3,423 (23.3) | 9,581 (42.1) |  |
| *5* | 48,371 (17.8) | 33,427 (16.5) | 10,177 (31.8) | 692 (4.7) | 4,075 (17.9) |  |
| *Unknown* | 71 (0.0) | 48 (0.0) | . (.) | 1 (0.0) | 22 (0.1) |  |
| **Stage summary** |  | | | | | <0.001 |
| *Localized* | 244,980 (89.9) | 182,222 (89.8) | 29,675 (92.6) | 12,915 (88) | 20,168 (88.7) |  |
| *Regional* | 27,500 (10.1) | 20,806 (10.2) | 2,364 (7.4) | 1,759 (12.0) | 2,571 (11.3) |  |
| **Residence type** |  | | | | | <0.001 |
| *Rural* | 3,971 (1.5) | 3,629 (1.8) | 283 (0.9) | 2 (0.0) | 57 (0.3) |  |
| *Urban* | 268,438 (98.5) | 199,351 (98.2) | 31,756 (99.1) | 14,671 (100) | 22,660 (99.7) |  |
| *Unknown* | 71 (0.0) | 48 (0.0) | . (.) | 1 (0.0) | 22 (0.1) |  |
| **Treatment type** |  | | | | | <0.001 |
| *None* | 73,269 (26.9) | 53,191 (26.2) | 10,179 (31.8) | 3,723 (25.4) | 6,176 (27.2) |  |
| *RT only* | 108,618 (39.9) | 80,298 (39.6) | 13,636 (42.6) | 6,055 (41.3) | 8,629 (37.9) |  |
| *Surgery only* | 78,234 (28.7) | 60,379 (29.7) | 6,771 (21.1) | 4,178 (28.5) | 6,906 (30.4) |  |
| *Surgery + RT* | 6,351 (2.3) | 4,643 (2.3) | 688 (2.1) | 409 (2.8) | 611 (2.7) |  |
| *Surgery + Unknown RT* | 1,131 (0.4) | 908 (0.4) | 99 (0.3) | 57 (0.4) | 67 (0.3) |  |
| *Unknown* | 4,715 (1.7) | 3,472 (1.7) | 649 (2.0) | 250 (1.7) | 344 (1.5) |  |
| *Unknown Surgery + RT* | 162 (0.1) | 137 (0.1) | 17 (0.1) | 2 (0.0) | 6 (0.0) |  |
| **Grade**^b^ |  |  |  |  |  | <0.001 |
| *Well differentiated* | 2,910 (1.1) | 2,104 (1.0) | 313 (1.0) | 153 (1.0) | 340 (1.5) |  |
| *Moderately differentiated* | 113,919 (41.8) | 85,926 (42.3) | 12,596 (39.3) | 5,445 (37.1) | 9,952 (43.8) |  |
| *Poorly differentiated* | 147,618 (54.2) | 109,123 (53.7) | 18,084 (56.4) | 8,651 (59.0) | 11,760 (51.7) |  |
| *Undifferentiated* | 661 (0.2) | 494 (0.2) | 83 (0.3) | 31 (0.2) | 53 (0.2) |  |
| *Unknown* | 7,372 (2.7) | 5,381 (2.7) | 963 (3.0) | 394 (2.7) | 634 (2.8) |  |
| Abbreviations: AAPI = Asian American/Pacific Islander; SES = socioeconomic status; RT = radiotherapy  P-values were obtained from Kruskal-Wallis test for continuous variable and Chi-square test for categorical variables.  ^a^Represents all Hispanic/Latino individuals from the study, including those reported as Hispanic/Latino, NOS.  ^b^Well differentiated, moderately differentiated, and poorly differentiated histologic grading correlate with Gleason scores 2-4, 5-6, and 7-10, respectively. | | | | | | |

| **Supplemental Table 4. Patient, tumor, and treatment characteristics divided by racial and/or ethnic subgroup for men ≥65 years old** | | | | | | | | |
| --- | --- | --- | --- | --- | --- | --- | --- | --- |
| *Variable* | *All* | *Non-Hispanic White* | *Mexican* | *Cuban* | *Puerto Rican* | *Dominican Republic* | *South or Central American* |  |
|  | *N (%)* | *N (%)* | *N (%)* | *N (%)* | *N (%)* | *N (%)* | *N (%)* | *p-value* |
| **Total patients** | 210,736 | 203,028 | 4,683 | 581 | 149 | 149 | 1,573 |  |
| **Age at diagnosis** | 72.7 ± 6.0 | 72.7 ± 6.0 | 72.1 ± 5.7 | 73.8 ± 5.9 | 72.3 ± 6.0 | 71.9 ± 5.5 | 71.8 ± 5.6 | <0.001 |
| **Marital status** |  | | | | | | | <0.001 |
| *Married* | 146,038 (69.3) | 140,637 (69.3) | 3,373 (72.0) | 405 (69.7) | 95 (63.8) | 95 (63.8) | 1,103 (70.1) |  |
| *Others* | 39,055 (18.5) | 37,359 (18.4) | 938 (20.0) | 137 (23.6) | 37 (24.8) | 37 (24.8) | 353 (22.4) |  |
| *Unknown* | 25,643 (12.2) | 25,032 (12.3) | 372 (7.9) | 39 (6.7) | 17 (11.4) | 17 (11.4) | 117 (7.4) |  |
| **Insurance status** |  | | | | | | | <0.001 |
| *Uninsured* | 601 (0.3) | 503 (0.2) | 43 (0.9) | 3 (0.5) | 2 (1.3) | 2 (1.3) | 43 (2.7) |  |
| *Any Medicaid* | 4,403 (2.1) | 3,142 (1.5) | 878 (18.7) | 48 (8.3) | 27 (18.1) | 27 (18.1) | 237 (15.1) |  |
| *Insured* | 139,820 (66.3) | 135,954 (67.0) | 2,279 (48.7) | 307 (52.8) | 86 (57.7) | 86 (57.7) | 811 (51.6) |  |
| *Unknown* | 65,912 (31.3) | 63,429 (31.2) | 1,483 (31.7) | 223 (38.4) | 34 (22.8) | 34 (22.8) | 482 (30.6) |  |
| **T stage** |  | | | | | | | <0.001 |
| *Tx* | 66 (0.0) | 63 (0.0) | 2 (0.0) | 1 (0.2) | . (.) | . (.) | . (.) |  |
| *T0* | 2 (0.0) | 2 (0.0) | . (.) | . (.) | . (.) | . (.) | . (.) |  |
| *T1* | 95,566 (45.3) | 92,147 (45.4) | 1,908 (40.7) | 297 (51.1) | 98 (65.8) | 98 (65.8) | 736 (46.8) |  |
| *T2* | 98,207 (46.6) | 94,645 (46.6) | 2,303 (49.2) | 252 (43.4) | 43 (28.9) | 43 (28.9) | 687 (43.7) |  |
| *T3* | 15,121 (7.2) | 14,501 (7.1) | 401 (8.6) | 25 (4.3) | 6 (4.0) | 6 (4.0) | 134 (8.5) |  |
| *T4* | 1,774 (0.8) | 1,670 (0.8) | 69 (1.5) | 6 (1.0) | 2 (1.3) | 2 (1.3) | 16 (1.0) |  |
| **N stage** |  | | | | | | | <0.001 |
| *Nx* | 2,917 (1.4) | 2,761 (1.4) | 77 (1.6) | 18 (3.1) | 8 (5.4) | 8 (5.4) | 37 (2.4) |  |
| *N0* | 205,215 (97.4) | 197,801 (97.4) | 4,514 (96.4) | 558 (96.0) | 139 (93.3) | 139 (93.3) | 1,507 (95.8) |  |
| *N1* | 2,604 (1.2) | 2,466 (1.2) | 92 (2.0) | 5 (0.9) | 2 (1.3) | 2 (1.3) | 29 (1.8) |  |
| **M stage** |  | | | | | | |  |
| *M0* | 210,736 (100) | 203,028 (100) | 4,683 (100) | 581 (100) | 149 (100) | 149 (100) | 1,573 (100) |  |
| **SES composite score** |  | | | | | | | <0.001 |
| *1* | 44,103 (20.9) | 43,121 (21.2) | 408 (8.7) | 74 (12.7) | 37 (24.8) | 37 (24.8) | 259 (16.5) |  |
| *2* | 45,239 (21.5) | 44,200 (21.8) | 503 (10.7) | 94 (16.2) | 35 (23.5) | 35 (23.5) | 257 (16.3) |  |
| *3* | 43,532 (20.7) | 42,525 (20.9) | 687 (14.7) | 40 (6.9) | 18 (12.1) | 18 (12.1) | 133 (8.5) |  |
| *4* | 43,473 (20.6) | 39,707 (19.6) | 2,267 (48.4) | 363 (62.5) | 57 (38.3) | 57 (38.3) | 888 (56.5) |  |
| *5* | 34,341 (16.3) | 33,427 (16.5) | 818 (17.5) | 10 (1.7) | 2 (1.3) | 2 (1.3) | 36 (2.3) |  |
| *Unknown* | 48 (0.0) | 48 (0.0) | . (.) | . (.) | . (.) | . (.) | . (.) |  |
| **Stage summary** |  | | | | | | | <0.001 |
| *Localized* | 188,932 (89.7) | 182,222 (89.8) | 4,021 (85.9) | 537 (92.4) | 140 (94.0) | 140 (94.0) | 1,372 (87.2) |  |
| *Regional* | 21,804 (10.3) | 20,806 (10.2) | 662 (14.1) | 44 (7.6) | 9 (6.0) | 9 (6.0) | 201 (12.8) |  |
| **Residence type** |  | | | | | | |  |
| *Rural* | 3,640 (1.7) | 3,629 (1.8) | 11 (0.2) | . (.) | . (.) | . (.) | . (.) |  |
| *Urban* | 207,048 (98.2) | 199,351 (98.2) | 4,672 (99.8) | 581 (100) | 149 (100) | 149 (100) | 1,573 (100) |  |
| *Unknown* | 48 (0.0) | 48 (0.0) | . (.) | . (.) | . (.) | . (.) | . (.) |  |
| **Treatment type** |  | | | | | | | <0.001 |
| *None* | 54,940 (26.1) | 53,191 (26.2) | 1,099 (23.5) | 119 (20.5) | 32 (21.5) | 32 (21.5) | 350 (22.3) |  |
| *RT only* | 83,412 (39.6) | 80,298 (39.6) | 1,716 (36.6) | 303 (52.2) | 80 (53.7) | 80 (53.7) | 637 (40.5) |  |
| *Surgery only* | 62,826 (29.8) | 60,379 (29.7) | 1,645 (35.1) | 129 (22.2) | 27 (18.1) | 27 (18.1) | 491 (31.2) |  |
| *Surgery + RT* | 4,901 (2.3) | 4,643 (2.3) | 152 (3.2) | 18 (3.1) | 5 (3.4) | 5 (3.4) | 61 (3.9) |  |
| *Surgery + Unknown RT* | 936 (0.4) | 908 (0.4) | 18 (0.4) | 2 (0.3) | 1 (0.7) | 1 (0.7) | 5 (0.3) |  |
| *Unknown* | 3,581 (1.7) | 3,472 (1.7) | 51 (1.1) | 10 (1.7) | 4 (2.7) | 4 (2.7) | 29 (1.8) |  |
| *Unknown Surgery + RT* | 140 (0.1) | 137 (0.1) | 2 (0.0) | . (.) | . (.) | . (.) | . (.) |  |
| **Grade**^a^ |  |  |  |  |  |  |  | <0.001 |
| *Well differentiated* | 2,234 (1.1) | 2,104 (1) | 81 (1.7) | 14 (2.4) | 1 (0.7) | 1 (0.7) | 24 (1.5) |  |
| *Moderately differentiated* | 89,177 (42.3) | 85,926 (42.3) | 1,915 (40.9) | 250 (43.0) | 67 (45.0) | 67 (45.0) | 692 (44.0) |  |
| *Poorly differentiated* | 113,215 (53.7) | 109,123 (53.7) | 2,556 (54.6) | 298 (51.3) | 75 (50.3) | 75 (50.3) | 803 (51.0) |  |
| *Undifferentiated* | 511 (0.2) | 494 (0.2) | 9 (0.2) | 2 (0.3) | . (.) | . (.) | 4 (0.3) |  |
| *Unknown* | 5,599 (2.7) | 5,381 (2.7) | 122 (2.6) | 17 (2.9) | 6 (4.0) | 6 (4.0) | 50 (3.2) |  |
| Abbreviations: SES = socioeconomic status; RT = radiotherapy  P-values were obtained from Kruskal-Wallis test for continuous variable and Chi-square test for categorical variables.  ^a^Well differentiated, moderately differentiated, and poorly differentiated histologic grading correlate with Gleason scores 2-4, 5-6, and 7-10, respectively. | | | | | | | | |
